# Provincial Variation in Adherence to Breast Cancer Screening in Canada: Evidence From the Canadian Partnership for Tomorrow's Health

**DOI:** 10.1002/cam4.70543

**Published:** 2025-03-13

**Authors:** M. Darvishian, A. Moustaqim‐Barrette, P. Awadalla, P. Bhatti, P. Broet, R. A. Murphy, K. Skead, R. Urquhart, J. Vena, T. J. B. Dummer

**Affiliations:** ^1^ School of Population and Public Health University of British Columbia Vancouver British Columbia Canada; ^2^ Faculty of Medicine, McGill University Montreal Quebec Canada; ^3^ Ontario Institute for Cancer Research Toronto Ontario Canada; ^4^ Department of Molecular Genetics University of Toronto Toronto Ontario Canada; ^5^ Cancer Control Research, British Columbia (BC) Cancer Vancouver British Columbia Canada; ^6^ Department of Preventive and Social Medicine, École de Santé Publique Université de Montréal Montreal Quebec Canada; ^7^ Research Centre, CHU Sainte Justine Montreal Quebec Canada; ^8^ Department of Community Health and Epidemiology Dalhousie University Halifax Nova Scotia Canada; ^9^ Alberta Health Services, Alberta's Tomorrow Project Cancer Research & Analytics, Cancer Care Alberta Edmonton Alberta Canada

**Keywords:** breast cancer, cancer prevention, screening

## Abstract

**Background:**

Breast cancer is the most commonly diagnosed cancer among women in Canada. Screening is effective in reducing breast cancer mortality through early cancer detection. However, data on individual social and medical characteristics contributing to variation in adherence to screening is limited.

**Methods:**

Using multivariable logistic regression, we analyzed self‐reported questions on engagement in screening mammography from five regions of the Canadian Partnership for Tomorrow's Health (CanPath), including the BC Generations Project (BCGP), Alberta's Tomorrow Project (ATP), the Ontario Health Study (OHS), Quebec's CARTaGENE, and the Atlantic Partnership for Tomorrow's Health Study (Atlantic PATH).

**Results:**

The study population included 79,986 and 46,907 individuals aged 50–74 and 40–49 years at study enrollment, respectively. Most participants self‐reported undergoing screening mammography less than 2 years from study enrollment, ranging from 77.8% in OHS to 86.3% in BCGP. Factors significantly associated with a lower odd of ever undergoing screening mammography were lower household income, being single/never married, current daily smoking, poor self‐perceived health, no history of breast feeding, and ≥ 24 months since last routine medical check‐up by a doctor or nurse. Among women aged 40–49 years with a first‐degree family history of breast cancer (*N* = 4212 [8.9%]), the likelihood of ever being screened varied by region and was significantly lower among individuals with post menopause and more than 12 months since last medical check‐up.

**Conclusion:**

Factors associated with screening adherence that were identified in this study namely household income, self‐perceived health, and routine medical check‐ups should be considered as potential factors for targeting undeserved communities and increasing engagement in screening at both provincial and national levels. The observed variation in mammography among women aged 40 to 49 years with family history of breast cancer, may inform the current guidelines for potential benefits of early screening initiation.

## 1 Introduction

Breast cancer is the most commonly diagnosed cancer and the second leading cause of cancer‐related death among women in Canada [1, 2]. Breast cancer screening, as a secondary prevention strategy, has been shown to be effective in reducing cancer mortality through early cancer detection [3]. Evidence from observational studies show a 25% to 31% reduction in risk of breast cancer mortality among women aged 50 to 69 years [4].

Established guidelines by the Canadian Task Force on Preventive Health Care (CTFPHC) recommend biennial mammography for average risk women aged 50–74 years [5, 6]. In Canada, organized screening uses a systematic approach for the identification and invitation of the screening eligible population, recall after a normal or benign screening, and regular monitoring and evaluation. Important components of the organized approach to screening in Canada include providing consistent and high‐quality services, monitoring of screening program elements, integration of screening within the cancer care spectrum, as well as high enrollment and participation [7].

Although individuals at high risk (e.g., family history of breast cancer and genetic mutations) have a greater lifetime risk of developing breast cancer, currently there are no national guidelines, and screening protocols vary across jurisdictions [8]. For instance, although about 10% of breast cancer‐related deaths occurs among women aged 40–49 years, the risk of false positive and over diagnosis among this population has resulted in either excluding these individuals from routine screening policies or restricting the mammography only to those with family history of breast cancer in most Canadian jurisdictions [1, 9]. Nevertheless, although the risk of breast cancer is two times higher among women with affected first‐degree relative, engagement in mammographic screening among this population remains suboptimal [10, 11].

In Canada, preventive healthcare services, including screening programs, are part of publicly funded healthcare [12]. However, the most recent Canadian Community Health Survey indicates about 78% of Canadian women aged 50–74 years reported a history of breast cancer screening with mammography over the last 3 years [11]. However, an assessment of variation in screening uptake within and between provinces is needed [12]. In a recently conducted study, the inter‐ and intraprovincial variation in screen‐detected breast cancer cases varied from 42% to 52% among women aged 50–69 years [12]. While the observed within provincial variation was largely related to age‐group screening eligibility, the between province variation was associated with differences in rural/urban residence and income [12]. However, this study did not assess the potential impact of individual factors, known to be associated with breast cancer and health‐seeking behavior [11], such as education, ethnicity, history of pregnancy, and breast feeding as well as use of hormone replacement therapy and contraceptives.

In the current study, we used data from CanPath (the Canadian Partnership for Tomorrow's Health) [13] to identify factors associated with engagement in screening mammography and to estimate the potential variation in screening uptake across regional CanPath cohorts among eligible women (i.e., aged 50–74 years). We further assessed the potential factors associated with ever being screened among women aged 40 to 49 years with family history of breast cancer.

## 2 Methods

### 2.1 Study Population

CanPath participants were recruited from 2008 to 2016 from five regional cohorts namely the BC Generations Project (BCGP), Alberta's Tomorrow Project (ATP), the Ontario Health Study (OHS), Quebec's CARTaGENE, and the Atlantic Partnership for Tomorrow's Health Study (Atlantic PATH). Using a Health and Lifestyle Questionnaire (HLQ), data on following social and medical determinants of health at study enrollment were collected: age, sex, education, country of birth, race, marital status, income, self‐perceived health, family history of cancer and chronic diseases, physical activity, smoking status, number of pregnancies, breast feeding history, and cancer screening history. For the current analysis, the study population was restricted to women aged 40–74 years with no prior diagnosis of breast cancer. Individuals with missing information on age, cancer status, and/or type and unknown history of breast cancer screening were excluded.

### 2.2 Screening Status and Risk Factors

To examine engagement in screening mammography, the HLQ included two questions on lifetime history of breast cancer screening, as well as the timing of the last mammogram at study enrollment (i.e., less than 6 months ago, 6 months to less than 1 year ago, 1 year to less than 2 years ago, 2 years to less than 3 years ago, 3 years to less than 5 years ago, and 5 or more years ago). In this study, we categorized breast cancer screening status as “never screened” if participants reported no previous history of mammography and “ever screened” if participants reported any history of breast cancer screening at study enrollment. We further categorized ever screening status as history of screening “less than two years” if participants reported history of mammography less than 2 years ago, and “more than two years” if participants reported history of mammography more than 2 years ago. In order to examine the impact of family history of breast cancer, adherence to screening mammography was assessed separately among participants with a first‐degree family history of breast cancer [8] (i.e., first‐degree family history) and screening eligible women aged 50–74 without family history of breast cancer (i.e., average risk). As a sensitivity analysis, we also assessed ever versus never screening status among women aged 40 to 49 years with and without family history of breast cancer.

### 2.3 Statistical Analysis

Sociodemographic characteristics of participants stratified by study cohort at enrollment are presented as counts and percentages.

Multivariable logistic regression models were used to assess adherence to the screening mammography among two groups of ever versus never screened individuals as well as participants with history of breast cancer screening “less than two years” versus “more than two years” ago.

In the models, only variables selected through backward selection were included to evaluate the association between breast cancer screening and potential predictors. Associations were estimated as odds ratios (ORs) with 95% confidence intervals (95% CI).

Variables included in the models were sex, age (i.e., 40–49, 50–59, 60–69, and 70–74 years), total annual household income (i.e., <$50,000, $50,000–99,999, ≥$100,000), education (i.e., no education or less than high school, trade, technical school or diploma from a community college, university certificate below bachelor's level, bachelor's degree, and graduate degree), marital status (i.e., married or living with a partner, divorced, widowed, separated, single/never married), ethnic background (i.e., white, other), first language (i.e., English, French, other), perception of health (i.e., poor, fair, good, very good, and excellent), country of birth (i.e., Canada, other), smoking status (i.e., never smoked at least 100 cigarettes, past smoker (ever smoked at least 100 cigarettes), current occasional smoker, current daily smoker), and level of physical activity (i.e., low, moderate, or high). Models were also adjusted for the presence of comorbidities (defined as any occurrence of at least one of the following conditions: asthma, arthritis or rheumatism, high blood pressure, migraine headaches, chronic bronchitis or emphysema, sinusitis, diabetes, epilepsy, heart disease, cancer, stomach or intestinal ulcers, effects of a stroke, urinary incontinence, bowel disorders, Alzheimer's disease or dementia, cataracts, glaucoma, and thyroid dysfunction), time since last routine medical check‐up by a doctor or a nurse (< 12 months, ≤ 12 to < 24 months, ≥ 24 months), number of pregnancies (0, 1, 2, ≥ 3), total lifetime duration of breast feeding (0, ≤ 12 months, > 12 months), ever use of hormone replacement therapy (HRT) (no/yes), ever use of hormone fertility treatment (HFT) (no/yes), ever use of contraceptives (no/yes), and menopause (no/yes). Due to the self‐reported nature of the data, missing values in this study were categorized as “unknown” and were included in the analysis.

All analyses were performed using SAS version 9.4 (Cary, NC, USA). Ethical approval was provided by the Health Research Ethics Board, University of British Columbia.

## 3 Results

### 3.1 Sociodemographic Characteristics at Study Enrollment

**FIGURE 1:**
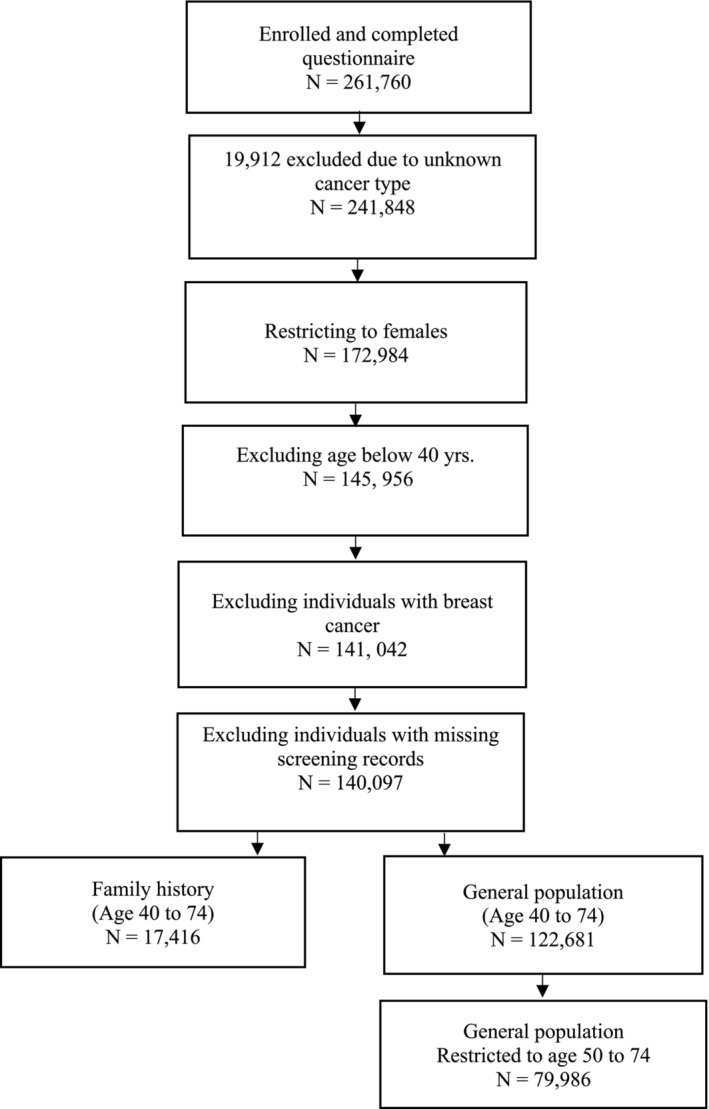
Study flow diagram.

From a total of 261,760 respondents at enrollment in CanPath, 79,986 average risk individuals aged 50–74 years, including 11,155 (14.0%) from BCGP, 13,374 (16.7%) from ATP, 36,871 (46.1%) from OHS, 11,898 (14.9%) from CARTaGENE, and 6688 (8.4%) from Atlantic PATH, met the inclusion criteria (Table 1 and Figure 1). Additionally, 17,416 individuals aged 40–74 years with a family history of breast cancer, including 2355 (13.5%) from BCGP, 3324 (19.1%) from ATP, 7986 (45.9%) from OHS, 1974 (11.3%) from CARTaGENE, and 1777 (10.2%) from Atlantic PATH, were included in the study (Table 2 and Figure 1).

**TABLE 1:** Summary characteristics of Canadian Partnership for Tomorrow's Health (CanPath) by region.

	Overall *N* (%)	Atlantic PATH *N* (%)	ATP *N* (%)	BCGP *N* (%)	CARTaGENE *N* (%)	OHS *N* (%)
Total	79,986 100.0	6688 8.4	13,374 16.7	11,155 14.0	11,898 14.9	36,871 46.1
Lifetime breast cancer screening						
Never	3780 4.73	299 4.47	342 2.56	294 2.64	659 5.54	2186 5.93
Ever	76,206 95.27	6389 95.53	13,032 97.44	10,861 97.36	11,239 94.46	34,685 94.07
Breast cancer screening status						
Never	3780 4.73	299 4.47	342 2.56	294 2.64	659 5.54	2186 5.93
Less than 2 years	63,994 80.00	5303 79.29	11,076 82.82	9621 86.25	9307 78.22	28,687 77.81
More than 2 years	12,212 15.27	1086 16.23	1956 14.62	1240 11.11	1932 16.24	5998 16.26
Age						
50–59	47,435 59.30	4153 62.09	7732 57.81	5557 49.82	7519 63.20	22,474 60.95
60–69	30,170 37.72	2466 36.87	5128 38.34	5584 50.00	4339 36.47	12,653 34.32
70–74	2381 2.98	69 1.03	514 3.84	14 0.12	40 0.33	1744 4.73
Household income						
< $50,000	20,394 25.50	1838 27.48	2945 22.02	2878 25.80	3783 31.79	8950 24.27
$50,000–$99,999	27,490 34.37	2582 38.60	4405 32.94	4233 37.94	3990 33.53	12,280 33.30
≥ $100,000	22,527 28.16	1687 25.22	4961 37.09	3229 28.95	2596 21.82	10,054 27.27
Unknown	9575 11.97	581 8.69	1063 7.95	815 7.31	1529 12.85	5587 15.53
Education						
No education, or less than high school	20,346 25.44	1546 23.11	3633 27.16	2467 22.11	3060 22.72	9640 26.15
Trade, technical school or diploma from community college	27,020 33.78	2628 39.29	4850 36.26	3703 33.20	3894 37.73	11,945 32.40
University certificate below bachelor's	4506 5.63	385 5.76	735 5.50	761 6.82	993 8.35	1632 4.43
Bachelor's degree	17,718 22.15	1351 20.20	2853 21.33	2542 22.79	2483 20.87	8489 23.02
Graduate degree	9604 12.01	754 11.27	1298 9.70	1625 14.57	1210 10.17	4717 12.79
Unknown	792 0.99	24 0.36	5 0.04	57 0.51	258 2.17	448 1.21
First language learned						
English	57,025 71.30	5725 85.60	11,819 88.37	9688 86.85	814 6.84	28,979 78.60
French	14,501 18.13	876 13.10	523 3.91	316 2.83	10,047 84.44	2739 7.43
Other	8460 10.57	87 1.30	1032 7.72	1151 10.32	1037 8.71	5153 13.97
Marital status						
Married or living with a partner	53,925 67.42	5078 75.93	9673 72.33	7848 70.35	7316 61.49	24,010 65.12
Divorced	11,584 14.48	660 9.87	1834 13.71	1529 13.70	1998 16.79	5563 15.09
Widowed	4902 6.13	406 6.07	878 6.56	614 5.50	564 4.74	2440 6.62
Separated	3020 3.78	194 2.90	295 2.21	324 2.90	419 3.52	1788 4.85
Single, never married	5807 7.26	326 4.87	691 5.17	796 7.14	1358 11.41	2636 7.15
Unknown	748 0.03	24 0.36	3 0.02	44 0.39	243 2.04	434 1.17
Self‐perceived health						
Poor	1396 1.74	65 0.97	85 0.63	106 0.95	233 1.96	907 2.46
Fair	6791 8.49	496 7.42	694 5.19	670 6.00	1266 10.64	3665 9.94
Good	25,336 31.68	2019 30.19	3931 29.39	3085 27.66	5177 43.51	11,124 30.17
Very good	32,593 40.75	3000 44.86	6246 46.70	4841 43.40	3780 31.77	14,726 39.94
Excellent	13,432 16.79	1100 16.45	2417 18.07	2393 21.45	1130 9.50	6392 17.34
Unknown	438 0.55	8 0.12	1 0.01	60 0.54	312 2.62	57 0.15
Country of birth						
Canada	64,810 81.03	6294 94.11	11,526 86.18	8410 75.39	10,648 89.49	27,932 75.76
Other	15,176 18.97	394 5.89	1848 13.82	2745 24.61	1250 10.51	8939 24.24
Ethnicity						
White	62,062 77.59	5617 83.98	8313 62.16	9542 85.54	8823 74.15	29,767 80.73
Other	17,924 22.41	1071 16.02	5061 37.84	1613 14.46	3075 25.85	7104 19.27
Physical activity level						
Low	17,782 22.23	1999 29.89	1914 14.31	1408 12.62	2557 21.49	9904 26.86
Moderate	22,461 28.08	2034 30.41	3136 23.45	2568 23.02	3743 31.46	10,980 29.78
High	24,912 31.14	2337 34.94	3954 29.56	3586 32.15	3735 31.39	11,300 30.65
Unknown	14,831 18.54	318 4.75	4370 32.68	3593 32.21	1863 15.66	4687 12.71
Smoking status						
Never smoked at least 100 cig	37,887 47.37	3114 46.56	6779 50.69	5713 51.21	4696 39.47	17,585 47.69
Past smoker (ever at least 100 cigarettes)	30,929 38.67	2835 42.39	4523 33.82	4767 42.73	5032 42.29	13,772 37.35
Current occasional smoker	1542 1.93	130 1.94	149 1.11	141 1.26	407 3.42	715 1.94
Current daily smoker	6905 8.63	522 7.80	811 6.06	414 3.71	1491 12.53	3667 9.95
Unknown	2723 3.40	87 1.30	1112 8.31	120 1.08	272 2.29	1132 3.07
Presence of comorbidity						
0	28,207 35.26	2533 37.87	5110 38.21	3808 34.14	4172 35.06	12,584 34.13
1	23,653 29.57	2268 33.91	4693 35.09	3137 28.12	2173 18.26	11,382 30.87
2	13,657 17.07	1294 19.35	2490 18.62	1674 15.00	1561 13.12	6638 18.00
3	4695 5.87	443 6.62	800 5.98	541 4.85	424 3.56	2487 6.74
4	2132 2.67	115 1.72	222 1.66	298 2.67	551 4.63	946 2.57
5	7587 9.48	26 0.39	55 0.41	1669 14.96	3017 25.36	2820 7.65
Unknown	55 0.07	9 0.13	4 0.03	28 0.25	0 0.00	14 0.04
Time Since last routine medical check‐up by a doctor or a nurse						
< 12 months	54,953 68.70	4727 70.68	9361 69.99	6984 62.61	8836 74.26	25,045 67.93
≤ 12 to < 24 months	15,751 19.69	1065 15.92	2584 19.32	2539 22.76	1731 14.55	7832 21.24
≥ 24 months	7859 9.83	729 10.90	983 7.35	1422 12.75	974 8.19	3751 10.17
Unknown	1423 1.78	167 2.50	446 3.33	210 1.88	357 3.00	243 0.66
Pregnancy						
0	11,004 13.76	800 11.96	1531 11.45	1751 15.70	1649 13.86	5273 14.30
1	9110 11.39	745 11.14	1194 8.93	1329 11.91	1679 14.11	4163 11.29
2	24,186 30.24	2234 33.40	4031 30.14	3287 29.47	3574 30.04	11,060 30.00
≥ 3	34,877 43.60	2881 43.08	6590 49.27	4717 42.29	4838 40.66	15,851 42.99
Unknown	809 1.01	28 0.42	28 0.42	71 0.63	158 1.33	524 1.42
Total lifetime duration of breast feeding						
0	18,660 23.33	2169 32.43	2832 21.17	1659 14.87	5203 43.73	6797 18.43
≤ 12 months	27,533 34.42	2309 34.52	5071 37.92	3905 35.01	3377 28.38	12,871 34.91
> 12 months	15,993 19.99	1118 16.72	3841 28.72	3220 28.86	1164 9.78	6650 18.04
Unknown	17,800 22.25	1092 16.33	1630 12.19	2371 21.25	2154 18.10	10,553 28.62
Ever use of hormone replacement therapy						
No	49,622 62.04	3912 58.49	7820 58.47	6130 54.95	7624 64.07	24,136 65.46
Yes	29,172 36.47	2211 33.06	5539 41.42	4915 44.06	4016 33.75	12,491 33.88
Unknown	1192 1.49	565 8.45	15 0.11	110 0.99	258 2.17	244 0.66
Ever use of hormone fertility treatment						
No	73,394 91.76	6383 95.44	12,707 95.01	10,552 94.59	11,109 93.37	32,643 40.81
Yes	4155 5.19	249 3.72	647 4.84	531 4.76	633 5.32	2095 5.68
Unknown	2437 3.05	56 0.84	20 0.15	72 0.65	156 1.31	2133 5.79
Ever use of contraceptives						
No	10,282 12.85	699 10.45	1501 11.22	1098 9.84	1916 16.10	5068 13.74
Yes	69,213 86.53	5965 89.19	11,866 88.72	10,005 89.69	9824 82.57	31,553 85.58
Unknown	491 0.62	24 0.36	7 0.05	52 0.47	158 1.33	250 0.68
Menopause						
No	12,820 16.03	1149 17.18	2058 15.39	1549 13.89	2207 18.55	5857 15.88
Yes	65,939 82.44	5439 81.32	11,155 83.41	9422 84.46	9365 78.72	30,558 82.88
Unknown	1227 1.53	100 1.49	161 1.20	184 1.65	326 2.74	456 1.24

**TABLE 2:** Summary characteristics of Canadian Partnership for Tomorrow's Health (CanPath), individuals with family history of breast cancer, by region.

	Overall *N* (%)	Atlantic PATH *N* (%)	ATP *N* (%)	BCGP *N* (%)	CARTaGENE *N* (%)	OHS *N* (%)
Total	17,416	1777	3324	2355	1974	7986
Lifetime breast cancer screening						
Never	764 4.39	66 3.71	85 2.56	57 2.42	99 5.02	457 5.72
Ever	16,652 95.61	1711 96.29	3239 97.44	2298 97.58	1875 94.98	7529 94.28
Breast cancer screening status						
Never	764 4.39	66 3.71	85 2.56	57 2.42	99 5.02	457 5.72
Less than 2 years	14,649 84.11	1533 86.27	2914 87.67	2123 90.15	1606 81.36	6473 81.05
More than 2 years	2003 11.50	178 10.02	325 9.78	175 7.43	269 13.63	1056 13.22
Age						
40–49	4212 24.18	435 24.48	713 21.45	425 18.05	450 22.80	2189 27.41
50–59	7147 41.04	740 41.64	1444 43.44	897 38.09	808 40.93	3258 40.80
60–69	5578 32.03	588 33.09	1035 31.14	1032 43.82	709 35.92	2214 27.72
70–74	479 2.75	14 0.79	132 3.97	1 0.04	7 0.35	325 4.07
Household income						
< $50,000	4086 23.46	434 24.42	626 18.83	549 23.31	690 9.37	1787 12.26
$50,000–$99,999	6009 34.50	669 37.65	1046 31.47	914 38.81	663 34.95	2717 22.38
≥ $100,000	5576 32.02	510 28.70	1390 41.82	737 31.30	436 33.59	2503 34.02
Unknown	1745 10.02	164 9.23	262 7.88	155 6.58	185 22.09	979 31.34
Education						
No education, or less than high school	4146 23.81	371 20.88	861 25.90	541 22.97	547 0.56	1826 22.87
Trade, technical school or diploma from community college	6007 34.49	687 38.66	1246 37.49	789 33.50	638 27.71	2647 33.15
University certificate below bachelor's	931 5.35	104 5.85	178 5.36	137 5.82	187 32.32	325 4.07
Bachelor's degree	4010 23.02	388 21.83	713 21.45	546 23.18	389 9.47	1974 24.72
Graduate degree	2198 12.62	222 12.49	325 9.78	321 13.63	202 19.71	1128 14.12
Unknown	124 0.71	5 0.28	1 0.03	21 0.89	11 10.23	86 1.08
First language learned						
English	13,254 76.10	1540 86.66	2967 89.26	2091 88.79	118 5.98	6538 81.87
French	2889 16.59	220 12.38	156 4.69	74 3.14	1750 88.65	689 8.63
Other	1273 7.31	17 0.96	201 6.05	190 8.07	106 5.37	759 9.50
Marital status						
Married or living with a partner	12,160 69.82	1358 76.42	2520 75.81	1680 71.34	1234 62.51	5368 67.22
Divorced	2242 12.87	151 8.50	384 11.55	303 12.87	317 16.06	1087 13.61
Widowed	882 5.06	85 4.78	168 5.05	115 4.88	92 4.66	422 5.28
Separated	655 3.76	60 3.38	65 1.96	56 2.38	74 3.75	400 5.01
Single, never married	1395 8.01	119 6.70	187 5.63	188 7.98	253 12.82	648 8.11
Unknown	82 0.47	4 0.23	0 0.00	13 0.55	4 0.20	61 0.76
Self‐perceived health						
Poor	276 1.58	13 0.73	18 0.54	22 0.38	30 1.52	193 2.42
Fair	1423 8.17	130 7.32	158 4.75	130 0.93	243 12.31	762 9.54
Good	5371 30.84	556 31.29	942 28.34	654 5.52	870 44.07	2349 29.41
Very good	7245 41.60	787 44.29	1587 47.74	1074 27.77	632 32.02	3165 39.63
Excellent	3063 17.59	288 16.21	618 18.59	466 45.61	179 9.07	1512 18.93
Unknown	38 0.22	3 0.17	1 0.030	9 19.79	20 1.01	5 0.06
Country of birth						
Canada	14,728 84.57	1681 94.60	2908 87.49	1862 79.07	1799 91.13	6478 81.12
Other	2688 15.43	96 5.40	416 12.52	493 20.93	175 8.87	1508 18.88
Ethnicity						
White	13,616 78.18	1495 84.13	2178 65.52	1998 84.84	1294 65.55	6651 83.28
Other	3800 21.82	282 15.87	1146 34.48	357 15.16	680 34.45	1335 16.72
Physical activity level						
Low	3915 22.48	531 29.88	471 14.17	364 15.46	375 19.00	2174 27.22
Moderate	5189 29.79	549 30.89	861 25.90	688 29.21	630 31.91	2461 30.82
High	5747 33.00	619 34.83	1053 31.68	902 38.30	631 31.97	2542 31.83
Unknown	2565 14.73	78 4.39	939 28.25	401 17.03	338 17.12	809 10.13
Smoking status						
Never smoked at least 100 cigarettes	8513 48.88	863 48.57	1709 51.41	1210 51.38	809 40.98	3922 49.11
Past smoker (ever at least 100 cigarettes)	6609 37.95	727 40.91	1088 32.73	996 42.29	826 41.84	2972 37.22
Current occasional smoker	353 2.03	35 1.97	36 1.08	32 1.36	78 3.95	172 2.15
Current daily smoker	1476 8.48	130 7.32	193 5.81	89 3.78	249 12.61	815 10.21
Unknown	465 2.67	22 1.24	298 8.97	28 1.19	12 0.61	105 1.31
Presence of comorbidity						
0	6885 39.53	725 40.80	1390 41.82	966 41.02	787 39.87	3017 37.78
1	5361 30.78	616 34.67	1123 33.78	747 31.72	451 22.85	2424 30.35
2	2771 15.91	284 15.98	570 17.15	347 14.73	293 14.84	1277 15.99
3	972 5.58	118 6.64	180 5.42	109 4.63	67 3.39	498 6.24
4	403 2.31	23 1.29	50 1.50	38 1.61	70 3.55	222 2.78
5	1012 5.81	8 0.45	10 0.30	145 6.16	306 15.50	543 6.80
Unknown	12 0.07	3 0.17	1 0.03	3 0.13	0 0.00	5 0.06
Time since last routine medical check‐up by a doctor or a nurse						
< 12 months	11,955 68.64	1278 71.92	2380 71.60	1444 61.32	1406 71.23	5447 68.21
≤ 12 to < 24 months	3447 19.79	274 15.42	614 18.47	562 23.86	310 15.70	1687 21.12
≥ 24 months	1703 9.78	187 10.52	223 6.71	308 13.08	177 8.97	808 10.12
Unknown	311 1.79	38 2.14	107 3.22	41 1.74	81 4.10	44 0.55
Pregnancy						
0	2535 14.56	245 13.79	382 11.49	381 16.18	279 14.13	1248 15.63
1	1970 11.31	201 11.31	303 9.12	269 11.42	286 14.49	911 11.41
2	5248 30.13	585 32.92	999 30.05	710 30.15	620 31.41	2334 29.23
≥ 3	7545 43.32	738 41.53	1635 49.19	984 41.78	785 39.77	3403 42.61
Unknown	118 0.68	8 0.45	5 0.15	11 0.47	4 0.20	90 1.13
Total lifetime duration of breast feeding						
0	3615 20.76	503 28.31	639 19.22	358 15.20	768 38.91	1347 16.87
≤ 12 months	5834 33.50	591 33.26	1250 37.61	766 32.53	614 31.10	2613 32.72
> 12 months	3995 22.94	352 19.81	1030 30.99	751 31.89	209 10.59	1653 20.70
Unknown	3972 22.81	331 18.63	405 12.18	480 20.38	383 19.40	2373 29.71
Ever use of hormone replacement therapy						
No	12,118 69.58	1085 61.06	2274 68.41	1501 63.74	1413 71.58	5845 73.19
Yes	4960 28.48	433 24.37	1043 31.38	829 35.20	548 27.76	2107 26.38
Unknown	338 1.94	259 14.58	7 0.21	25 1.06	13 0.66	34 0.43
Ever use of hormone fertility treatment						
No	15,998 91.86	1694 95.33	3146 94.65	2222 94.35	1870 94.73	7066 88.48
Yes	999 5.74	66 3.71	172 5.17	114 4.84	102 5.17	545 6.82
Unknown	419 2.41	17 0.96	6 0.18	19 0.81	2 0.10	375 4.70
Ever use of contraceptives						
No	1990 11.43	176 9.90	351 10.56	211 8.96	293 14.84	959 12.01
Yes	15,375 88.28	1595 89.76	2971 89.38	2137 90.74	1677 84.95	6995 87.59
Unknown	51 0.29	6 0.34	2 0.06	7 0.30	4 0.20	32 0.40
Menopause						
No	5348 30.71	571 32.13	938 28.22	587 24.93	599 30.34	2653 33.22
Yes	11,860 68.10	1183 66.57	2356 70.88	1729 73.42	1347 68.24	5245 65.68
Unknown	208 1.19	23 1.29	30 0.90	39 1.66	28 1.42	88 1.10

Tables 1 and 2 summarize the sociodemographic characteristics of individuals with average risk and first‐degree family history of breast cancer, respectively. Overall, across all CanPath regions, in both groups most participants were married or living with a partner (family history: 69.8%), were white (family history: 78.2%), and were among never smokers (family history: 48.9%). Furthermore, greater proportions of participants had household incomes $50,000–99,999 (family history: 34.5%), with an education level of trade, technical school, or diploma from community college (family history: 34.5%), had very good self‐perceived health (family history: 41.6%), had a high level of physical activity (family history: 33.00%), with no comorbid conditions (family history: 39.5%), and with less than 12 months since last routine medical check‐up by a doctor or nurse (family history: 68.6%). Furthermore, in both first‐degree family history and average risk groups, history of at least three pregnancies (family history: 43.3%), ≤ 12 months lifetime duration of breast feeding (family history: 33.5%), ever use of contraceptives (88.3%), and menopause (family history: 68.1%) were reported more frequently compared to other categories. Among both average risk and family history groups across all provinces, about 95% of individuals reported history of lifetime breast cancer screening. In total, 80.0% of average risk group and 84.1% of family history group were among regular screening category.

Table 3 presents predictors of adherence to breast cancer screening among ever versus never screened individuals in average risk participants and individuals with family history group. Overall, compared to OHS (the CanPath region with the largest number of participants), the likelihood of being ever screened compared to being never screened was higher across all regions, ranging from 21% in CARTaGENE to 51% in ATP among average risk individuals (Table 3). Similar patterns were observed among individuals with family history of breast cancer. In both groups, lower household income, marital status other than married or living with a partner, current daily smoking status, no history of breast feeding or more than 12 months of lifetime breast feeding, and more than 12 months since last routine medical check‐up by a doctor or nurse, were significant barriers of ever being screened. Additionally, among average risk individuals, poor, fair, and good self‐perceived health status compared to excellent category were significant barriers of ever being screened with mammography (OR poor: 1.80; 1.44–2.26). In contracts, older age (average risk groups), presence of comorbidity, ever use of contraceptives in both groups, and ever use of HFT and HRT, were significantly associated with higher odds of ever being screened.

**TABLE 3:** Predictors of breast cancer screening comparing ever (reference category) versus never screening status among average risk population and individuals with family history of breast cancer aged 50–79 years.

Study population	Average risk *N* = 79,986	Family history *N* = 13,204
Variable	Odds ratio (95% CI)	Odds ratio (95% CI)
Region		
BCGP	0.74 (0.65–0.85)	0.68 (0.40–1.14)
Atlantic PATH	0.48 (0.43–0.54)	0.55 (0.36–0.86)
ATP	0.49 (0.43–0.55)	0.61 (0.39–0.95)
CARTaGENE	0.79 (0.67–0.84)	0.88 (0.50–1.55)
OHS	1.00	1.00
Age		
50–59	1.00	NA
60–69	0.51 (0.46–0.56)	NA
70–74	0.54 (0.41–0.71)	NA
Household income		
< $50,000	1.68 (1.51–1.86)	NA
$50,000–$99,999	1.20 (1.08–1.31)	NA
≥ $100,000	1.00	NA
Unknown	1.25 (1.10–1.42)	NA
First language learned		
English	NA	1.00
French	NA	0.93 (0.55–1.55)
Other	NA	1.75 (1.14–2.68)
Marital status		
Married or living with a partner	1.00	1.00
Divorced	1.15 (1.04–1.27)	1.45 (1.01–2.08)
Widowed	0.96 (0.82–1.14)	0.83 (0.43–1.60)
Separated	1.25 (1.08–1.46)	1.69 (0.96–2.99)
Single, never married	1.25 (0.99–1.28)	1.15 (0.97–2.38)
Unknown	1.24 (0.91–1.70)	3.51 (1.17–10.53)
Smoking status		
Never smoked at least 100 cigarettes	1.00	1.00
Past smoker (ever smoked at least 100 cigarettes)	0.98 (0.90–1.06)	1.46 (1.08–1.99)
Current occasional smoker	1.39 (1.12–1.72)	1.85 (0.78–4.38)
Current daily smoker	1.69 (1.53–1.88)	2.59 (1.76–3.83)
Unknown	1.02 (0.82–1.26)	0.66 (0.20–2.17)
Self‐perceived health		
Poor	1.80 (1.44–2.26)	NA
Fair	1.31 (1.30–1.51)	NA
Good	1.17 (1.04–1.30)	NA
Very good	1.03 (0.93–1.15)	NA
Excellent	1.00	NA
Unknown	1.29 (0.83–2.01)	NA
Presence of comorbidity		
0	1.00	1.00
1	0.78 (0.71–0.85)	0.71 (0.51–0.97)
2	0.76 (0.68–0.85)	0.51 (0.32–0.81)
3	0.74 (0.62–0.87)	0.44 (0.20–0.95)
4	0.72 (0.57–0.91)	0.89 (0.38–2.08)
5	0.69 (0.60–0.80)	0.77 (0.44–1.35)
Total lifetime duration of breast feeding		
0	1.33 (1.21–1.47)	NA
≤ 12 months	1.00	NA
> 12 months	1.27 (1.15–1.40)	NA
Unknown	1.29 (1.17–1.43)	NA
Ever use of contraceptives		
No	1.00	1.00
Yes	0.66 (0.60–0.73)	0.76 (0.53–1.09)
Unknown	0.94 (0.65–1.36)	4.40 (1.38–14.00)
Ever use of hormone fertility treatment		
No	1.00	NA
Yes	0.77 (0.65–0.92)	NA
Unknown	0.87 (0.69–1.08)	NA
Ever use of hormone replacement therapy		
No	1.00	NA
Yes	0.40 (0.36–0.44)	NA
Unknown	0.77 (0.59–1.00)	NA
Menopause		
No	1.00	1.00
Yes	0.48 (0.45–0.53)	0.61 (0.44–0.84)
Unknown	0.81 (0.64–1.02)	1.32 (0.54–3.24)
Time since last routine medical check‐up by a doctor or a nurse		
< 12 months	1.00	1.00
≤ 12 to < 24 months	1.34 (1.22–1.47)	1.72 (1.19–2.47)
≥ 24 months	4.26 (3.91–4.63)	5.70 (4.13–7.86)
Unknown	4.86 (4.13–5.72)	8.04 (4.69–13.79)

*Note:* Not applicable (NA): variables not selected through backward selection.

Table 4 displays the predictors of adherence to breast cancer screening among individuals with history of screening less than 2 years compared to more than 2 years ago. Among both groups, the likelihood of being screened less than 2 years ago was significantly higher in ATP compared with OHS. Overall, household incomes < $50,000, marital status other than being married or living with a partner, being a current or past smoker, low level of physical activity, poor self‐perceived health, and ≥ 24 months since last routine medical check‐up by a doctor or nurse were significantly associated with lower adherence to screening within the last 2 years. Among average risk individuals, ever use of HRT and HFT, and menopause were significantly associated with being screened less than 2 years ago.

**TABLE 4:** Predictors of breast cancer screening comparing less than 2 years (reference category) to more than 2 years.

Study population	Average risk *N* = 79,986	Family history *N* = 13,204
Variable	Odds Ratio (95% CI)	
Region		
BCGP	1.04 (0.96–1.12)	0.81 (0.66–1.01)
Atlantic PATH	0.95 (0.89–1.01)	0.94 (0.79–1.11)
ATP	0.60 (0.56–0.65)	0.58 (0.47–0.70)
CARTaGENE	0.98 (0.92–1.05)	1.01 (0.84–1.22)
OHS	1.00	1.00
Age		
50–59	1.00	1.00
60–69	0.96 (0.91–1.00)	0.98 (0.86–1.11)
70–74	1.30 (1.15–1.46)	1.77 (1.34–2.34)
Race/cultural origin		
White	1.00	NA
Other	1.07 (1.01–1.13)	NA
Household income		
< $50,000	1.24 (1.17–1.32)	1.20 (0.97–1.50)
$50,000–$99,999	1.08 (1.02–1.14)	1.28 (1.07–1.54)
≥ $100,000	1.00	1.00
Unknown	1.10 (1.02–1.19)	1.20 (0.97–1.50)
Country of birth		
Canada	1.00	NA
Other	1.14 (1.08–1.20)	NA
Marital status		
Married or living with a partner	1.00	1.00
Divorced	1.23 (1.16–1.31)	1.28 (1.07–1.51)
Widowed	1.23 (1.13–1.33)	1.14 (0.90–1.44)
Separated	1.31 (1.18–1.45)	1.46 (1.08–1.96)
Single, never married	1.13 (1.03–1.23)	1.25 (1.00–1.60)
Unknown	1.41 (1.14–1.74)	2.63 (1.27–5.42)
Smoking status		
Never smoked at least 100 cigarettes	1.00	1.00
Past smoker (ever smoked at least 100 cigarettes)	1.08 (1.04–1.13)	0.97 (0.85–1.11)
Current occasional smoker	1.10 (0.95–1.28)	1.42 (0.94–2.15)
Current daily smoker	1.53 (1.42–1.64)	1.61 (1.32–1.96)
Unknown	1.20 (1.07–1.35)	0.96 (0.66–1.40)
Physical activity level		
Low	1.06 (1.0–1.12)	NA
Moderate	0.95 (0.90–1.00)	NA
High	1.00	NA
Unknown	0.92 (0.86–0.98)	NA
Self‐perceived health		
Poor	1.77 (1.53–2.06)	1.81 (1.22–2.68)
Fair	1.50 (1.37–1.64)	1.27 (1.00–1.60)
Good	1.18 (1.11–1.27)	0.95 (0.80–1.14)
Very good	1.04 (0.97–1.10)	0.77 (0.64–0.91)
Excellent	1.00	1.00
Unknown	1.31 (0.97–1.77)	0.61 (0.17–2.21)
Presence of comorbidity		
0	1.00	NA
1	0.94 (0.90–0.99)	NA
2	0.98 (0.92–1.04)	NA
3	1.04 (0.95–1.14)	NA
4	1.10 (0.97–1.24)	NA
5	1.09 (1.00–1.18)	NA
Pregnancy		
0	0.81 (0.73–0.89)	NA
1	0.89 (0.83–0.96)	NA
2	0.89 (0.84–0.93)	NA
≥ 3	1.00	NA
Unknown	1.01 (0.82–1.26)	NA
Total lifetime duration of breast feeding		
0	1.04 (0.98–1.10)	NA
≤ 12 months	1.00	NA
> 12 months	1.09 (1.03–1.16)	NA
Unknown	1.17 (1.08–1.27)	NA
Ever use of contraceptives		
No	1.00	
Yes	0.93 (0.87–0.98)	NA
Unknown	0.70 (0.52–0.94)	NA
Ever use of hormone fertility treatment		
No	1.00	NA
Yes	0.85 (0.77–0.93)	NA
Unknown	0.86 (0.75–1.00)	NA
Ever use of hormone replacement therapy		
No	1.00	NA
Yes	0.80 (0.77–0.84)	NA
Unknown	0.93 (0.78–1.10)	NA
Menopause		
No	1.00	NA
Yes	0.92 (0.87–0.98)	NA
Unknown	0.97 (0.81–1.16)	NA
Time since last routine medical check‐up by a doctor or a nurse		
< 12 months	1.00	1.00
≤ 12 to < 24 months	1.71 (1.62–1.79)	1.66 (1.43–1.93)
≥ 24 months	5.92 (5.59–6.27)	6.73 (5.78–7.85)
Unknown	4.03 (3.56–4.57)	5.14 (3.70–7.15)

*Note:* Screening status among average risk population and individuals with family history of breast cancer, aged 50–79 years. Not applicable (NA): variables not selected through backward selection.

As a sensitivity analysis, the association between participant characteristics and screening patterns among individuals aged 40 to 49 was assessed. In total, 46,907 individuals, including 4212 (8.98%) with a family history of breast cancer and 42,695 (91.0%) with no family history of breast cancer were in their 40s at study enrollment (Table 5). Overall, across all provinces, 87% of individuals aged 40–49 years with a family history of breast cancer had engaged in breast cancer screening. In general, among individuals aged 40–49 years with family history of breast cancer, the likelihood of ever being screened in their 40s was significantly lower among participants who were post menopause (OR 1.83, 95% CI 1.34–2.50), and those who had gone more than 12 months since last routine medical check‐up by a doctor or nurse (OR 3.32, 95% CI 1.97–5.60). Among individuals in their 40s with no family history of breast cancer, household incomes < $50,000, divorced marital status, low level of physical activity, no history of breast feeding or more than 12 months of lifetime breast feeding, post menopause, and more than 12 months since last routine medical check‐up by a doctor or nurse were significantly associated with lower odds of ever being screened (results not shown).

**TABLE 5:** Breast cancer screening status among: (A) all women aged 40–49 years and (B) women aged 40–49 years with family history of breast cancer, by region.

	Overall *N* (%)	Atlantic PATH *N* (%)	ATP *N* (%)	BCGP *N* (%)	CARTaGENE *N* (%)	OHS *N* (%)
Breast cancer screening status among all participants						
Never	17,567 37.45	1436 34.52	1361 20.85	754 18.81	3946 46.38	10,070 42.49
Less than 2 years	20,858 44.47	2141 51.47	4182 64.05	2882 71.91	2668 31.36	8985 37.91
More than 2 years	8482 18.08	583 14.01	986 15.10	372 9.28	1894 22.26	4647 19.61
Total	46,907 100.00	4160 8.87	6529 13.92	4008 8.54	8508 18.14	23,702 50.53
B Breast cancer screening status among individuals with family history of breast cancer						
Never	531 12.61	49 11.26	57 7.99	31 7.29	66 14.67	328 14.98
Ever	3681 87.39	386 88.74	656 92.01	394 92.71	384 85.33	1861 85.02
Total	4212 100.00	435 10.33	713 16.93	425 10.09	450 10.68	2189 51.97

## 4 Discussion

In this study, adherence to breast cancer screening guidelines across eight provinces in Canada was assessed using data from five regional cohorts in CanPath. Overall, among average risk populations, the majority of participants were in the “ever screened” category. Similarly, a higher proportion of individuals with a family history of breast cancer were among ever screeners, ranging from 94% in OHS to 97% in BCGP. Among individuals aged 40–49 years with a first‐degree family history of breast cancer, the majority of participants had a history of ever being screened, ranging from 85% in CARTaGENE to 92% in BCGP. In multivariable regression analysis among average risk and participants with family history of breast cancer, the likelihood of being screened less than 2 years ago was significantly higher in ATP compared to OHS (the CanPath region with the largest number of participants). Overall, lower household income, current daily smoking, no history of breast feeding and more than 12 months since last routine medical check‐up by a doctor or nurse were among factors identified as significant barriers to screening uptake.

According to the Canadian Partnership Against Cancer (CPAC), adherence of 70% or higher to mammography screening is recommended as an effective strategy for reducing breast cancer‐related mortality in the general population [14]. Our findings show that the participation rate in all CanPath regions exceeded this target. In a recently conducted study in Alberta, adherence to screening at enrollment was 79%, which was in line with the 83% estimated rate in our study (1515). Nevertheless, in a population‐based cohort study conducted in Ontario in 2011, 64% of women aged 50–74 had history of at least one mammogram over the last 24 months, which was lower than the estimated 78% among average risk women in Ontario (OHS) in the current study [15]. Overall, the observed variation between regional cohorts in this study was minimal.

Similar to previously conducted studies, the present study found that several modifiable and non‐modifiable factors were significantly associated with regular or never screening. It has been shown that having a family doctor was significantly associated with regular screening [15, 16, 17]. Our study supports this finding as we observed a significant association between having a routine check‐up performed more than 12 months ago and never screening. Our results on the association of household income and regular screening echo the findings reported by two studies conducted in Ontario and Alberta [15, 18]. We further observed that racial origin other than white was significantly associated with episodic screening. However, due to limited diversity in race within CanPath participants, we were not able to further disaggregate “other” racial origin. In a study conducted by Woods et al. in British Columbia, significant variation in screening participation across country of birth was observed and Eastern European/Central Asian women showed low participation rate (38%) [19]. Overall, in the Woods study, participation rates for immigrant women from the most common birth countries, including China/Macau/Hong Kong/Taiwan (46%), India (45%), the Philippines (46%), and South Korea (39%), were lower than the nonimmigrant rates (51%) [19]. Hence, strategies for improving mammography adherence in women of racial and ethnic minorities may be required. These strategies could include reminders as well as educational interventions, taking into account the potential language barriers among minorities and immigrants [20].

Our finding on the potential associations between age and mammography partly supports the reported nonlinear association of increased screening adherence by age, followed by a decline among older participants [15, 21]. In the current study, compared with never being screened, the likelihood of regular screening was higher among individuals aged 60–69 years, in both average risk and first‐degree family history groups (Tables 3 and 4). Lower participation among the younger age in this analysis might be related to their lower self‐perceived risk of breast cancer which could potentially lead to underdiagnosis of cancer among this population and requires further investigation [12].

In Canada, some provinces and territories (i.e., British Columbia, Alberta, Nova Scotia, Prince Edward Island, and Northwest Territories) include women aged 40–49 years in their organized breast cancer screening program [1]. The potential risk of false positive and overdiagnosis of nonprogressive tumors might outweigh the benefits of screening among women in their 40s [1, 4, 9]. However, in a study conducted by Wilkinson et al., using Canadian Community Health Survey (CCHS) data between 2002 and 2007, the 10‐year breast cancer net survival rate was significantly higher in provinces including women aged 40–49 years in their screening program [1, 22]. In our study, we observed significant variation in adherence to screening among individuals aged 40–49 years with first‐degree family history of breast cancer, ranging from 85% in CARTaGENE to 92% in BCGP. Considering the reported benefits of screening namely cancer diagnosis at earlier stage and reduced cancer‐related death, future studies are required to further assess the risk–benefit of regular screening among women aged 40–49 years [23, 24, 25].

In a meta‐analysis conducted by Katapodi et al., the association between perceived risk of breast cancer and adherence to screening was influenced by a patient's physiological and psychological factors [26]. In a study conducted by Yuan et al., history of hypertension and hyperlipidemia were associated with increased mammography screening, while prior heart attack was associated with decreased annual mammographic screening [21]. In our study, the presence of comorbid conditions, especially having up to three conditions was associated with higher likelihood of adherence to screening, which could be related to more frequent medical check‐ups [27]. Future studies should explore to what extent adherence to regular screening could be influenced by the presence of comorbid conditions and estimate the “underutilization” of screening programs among healthy women [10, 26].

Evidence shows that current or recent use of progestogen‐only contraceptives are associated with a slight increase in breast cancer risk [28, 29]. Additionally, it is well known that prolonged estrogen exposure and combined HRT or estrogen‐only HRT usage for menopause are associated with increased risk of breast cancer [29, 30]. These findings highlight the importance of regular medical check‐ups as well as routine screening in this population. Similarly, in our study individuals with ever use of contraceptives, HRT, with menopause at study enrollment, or with higher risk of breast cancer, were more likely to regularly screen. However, HRT makes mammography screening less effective by adversely affecting the sensitivity and specificity of the test [30, 31]. Hence, factors including type of prescribed HRT and short‐term cessation of HRT therapy before mammography should be further explored in studies assessing the patterns of screening behavior among women on these therapies [32].

To our knowledge, this is the first Pan‐Canadian study to assess factors associated with breast cancer screening uptake in a general population cohort. The harmonized questionnaires in CanPath support the internal validity of the study and comparability of datasets across the different Canadian regions [13]. CanPath's large study sample, drawn from across eight provinces, enabled us to include participant‐level information, namely education, race/ethnicity, perceived health, cigarette smoking, presence of comorbidity conditions, and ever use of HFT and HRT. Hence, in this study, we were able not only to assess adherence to breast cancer screening recommendations but also to highlight the potential factors associated with adherence to regular screening, which can support future policy decision‐making. Despite these strengths, the following limitations should be considered while interpreting the results. First, the self‐reported nature of responses could potentially bias the derived estimates and associations, yet the observed variation is unlikely to be differential across study regions [33, 34]. Second, the generalizability of the findings could be affected by the voluntary enrollment of the participants in CanPath [35]. Furthermore, since data on follow‐up screening were not available, we were not able to assess the screening retention rates among participants, especially among individuals with first‐degree family history of breast cancer, in different regions. Finally, due to lack of information on genetic mutations, adherence to screening program among individuals at higher risk of breast cancer was solely assessed among participants with family history of breast cancer.

In conclusion, the majority of participants in the five regions of CanPath engaged in mammographic screening in alignment with current breast cancer screening recommendations, with slight variations among specific groups between regions. The potential factors associated with screening adherence that were identified, specifically household income, self‐perceived health, and routine medical check‐ups, should be considered as potential factors for targeting undeserved communities and improving engagement in screening at both provincial and national levels. The observed variation in mammography among women aged 40–49 years with family history of breast cancer may inform the current guidelines for potential benefits of early screening initiation.

## Author Contributions

**M. Darvishian:** conceptualization (equal), formal analysis (equal), methodology (equal), writing – original draft (equal). **A. Moustaqim‐Barrette:** conceptualization (equal), methodology (equal), writing – review and editing (equal). **P. Awadalla:** conceptualization (equal), writing – review and editing (equal). **P. Bhatti:** investigation (equal), methodology (equal), writing – review and editing (equal). **P. Broet:** conceptualization (equal), writing – review and editing (equal). **R. A. Murphy:** conceptualization (equal), methodology (equal), writing – review and editing (equal). **K. Skead:** conceptualization (equal), writing – review and editing (equal). **R. Urquhart:** conceptualization (equal), writing – review and editing (equal). **J. Vena:** conceptualization (equal), writing – review and editing (equal). **T. J. B. Dummer:** conceptualization (equal), methodology (equal), supervision (equal), writing – review and editing (equal).

## Ethics Statement

Ethical approval was provided by the Health Research Ethics Board, University of British Columbia. All participants in the CanPath provided written informed consent for participation in the study.

## Conflicts of Interest

The authors declare no conflicts of interest.

## Data Availability

The data analyzed in this study is subject to the following licenses/restrictions: “The data that support the findings of this study are available on request from CanPath—The Canadian Partnership for Tomorrow's Health (formerly CPTP). The data are not publicly available due to privacy or ethical restrictions.” Data access requests should be submitted to CanPath, https://canpath.ca/access‐process/.
